# A Randomized Cross-Over Trial Comparing the Effect of Intramuscular Versus Intranasal Naloxone Reversal of Intravenous Fentanyl on Odor Detection in Working Dogs

**DOI:** 10.3390/ani9060385

**Published:** 2019-06-22

**Authors:** Jennifer L. Essler, Paige G. Smith, Danielle Berger, Elizabeth Gregorio, M. Ross Pennington, Amanda McGuire, Kenneth G. Furton, Cynthia M. Otto

**Affiliations:** 1 Penn Vet Working Dog Center, School of Veterinary Medicine, University of Pennsylvania, Philadelphia, PA 19146, USA; paigegabrielles@gmail.com (P.G.S.); danieb@vet.upenn.edu (D.B.); eg2yt@virginia.edu (E.G.); cmotto@vet.upenn.edu (C.M.O.); 2 US Army Medical Research Institute of Chemical Defense, Aberdeen Proving Ground, Aberdeen Proving Ground, MD 21010, USA; mark.r.pennington.civ@mail.mil (M.R.P.); amanda.j.mcguire2.mil@mail.mil (A.M.); 3 Department of Chemistry and Biochemistry, International Forensic Research Institute, Florida International University, Miami, FL 33199, USA; furtonk@fiu.edu; 4 Department of Clinical Sciences and Advanced Medicine, School of Veterinary Medicine, University of Pennsylvania, Philadelphia, PA 19104, USA

**Keywords:** dogs, fentanyl, naloxone, odor detection, working dogs

## Abstract

**Simple Summary:**

The recent increase in fentanyl as an illicit street drug, paired with its powerful potency, has led to emergency personnel carrying naloxone, an opioid reversal agent, for the case of accidental exposures and overdoses in humans. Canine officers, if demonstrating intoxication from exposure to fentanyl, are consequently administered naloxone, however the effects of this treatment on the dogs’ scent detection are unknown. We tested the effects of intravenous and intranasal naloxone administration on dogs’ scent detection 2, 24, and 48 h after intravenous fentanyl sedation and naloxone reversal. We found no detectable influence of this fentanyl sedation and naloxone reversal on dogs’ abilities, regardless of whether they received intranasal or intramuscular naloxone. Results suggest there is no evidence that under these conditions, intravenous fentanyl followed by naloxone reversal impairs canine olfactory ability.

**Abstract:**

Fentanyl is a potent opioid used clinically as a pain medication and anesthetic but has recently seen a sharp rise as an illicit street drug. The potency of fentanyl means mucous membrane exposure to a small amount of the drug can expose first responders, including working canines, to accidental overdose. Naloxone, a fast-acting opioid antagonist administered intranasally (IN) or intramuscularly (IM) is currently carried by emergency personnel in the case of accidental exposure in both humans and canines. Despite the fact that law enforcement relies heavily on the olfactory abilities of canine officers, the effects of fentanyl exposure and subsequent reversal by naloxone on the olfactory performance of canines are unknown. In a block-randomized, crossover trial, we tested the effects of IN and IM naloxone on the abilities of working dogs to recognize the odor of Universal Detection Calibrant (UDC) prior to, and two, 24, and 48 h after intravenous fentanyl sedation and naloxone reversal. No detectable influence of fentanyl sedation and naloxone reversal on the dogs’ olfactory abilities was detected. We also found no difference in olfactory abilities when dogs received IN or IM naloxone. Together, results suggest no evidence that exposure to intravenous fentanyl followed by naloxone reversal impairs canine olfactory ability under these conditions.

## 1. Introduction

Fentanyl is an opioid first made and introduced into clinical medicine in 1960, where it was used as an intravenous anesthetic [1]. An increase in the number of fentanyl-related deaths has been observed with the increase in ways the drug can be administered [1,2,3]. This increase in fentanyl overdoses has been attributed to misuse, inappropriate prescribing, and illicit use and abuse of the drug. Additionally, fentanyl has low production costs, and as such is frequently used in the production of other illicit drugs, including heroin, methylenedioxymethamphetamine (MDMA), and counterfeit OxyContin (oxycodone), a fact often not known by the end-user of these substances [4]. 

First responders to fentanyl-linked incidences are at risk of exposure if they come into contact with fentanyl across mucous membranes (oral, nasal, ocular). Fentanyl may be present in illicit drug laboratories or from spilt containers of powder. Because of the increasing rate of fentanyl use, and the high risk involved to users and first responders, first responders have now begun carrying and utilizing naloxone to reverse opioid overdoses [5,6]. Naloxone is an opioid antagonist that reverses opioid effects by blocking opioid receptors [7]; it is transported easily across the blood–brain barrier which leads to fast reversal of opioid effects [8]. Naloxone can be administered intramuscularly as well as intranasally, and both routes are used by laypersons as well as emergency personnel [5].

Canine handler teams can also be first responders to drug-related incidents, leading them into situations where they could be exposed to fentanyl. The canines are asked to actively sniff out drugs [9], which can put the dogs in positions where they may inhale drugs laced with fentanyl. As in humans, naloxone is also an accepted form of opioid reversal in dogs [10,11], and more canine handlers are now being trained to use naloxone in cases of accidental exposure to their dogs. 

Despite the concern of fentanyl exposure and subsequent naloxone administration in dogs, any effect on the dogs’ olfaction has not been studied. The acquisition of forensic evidence by law enforcement officers, including canines, is subject to scrutiny, and factors that reduce the reliability of the detection dog may be introduced in court [12,13]. Multiple factors may affect the olfactory performance of a dog, including breed [9], diet, and exercise [14], disease and certain medications [15] although many gaps in the research remain [16]. The only medications confirmed to diminish canine olfaction are the antibiotic, metronidazole [17] and the steroids (high doses of dexamethasone or hydrocortisone combined with deoxycorticosterone) [18]. A recent thesis found no effect of either isoflurane or Propofol, two anesthetics, on the olfactory ability of odor detection dogs [19]. No studies thus far have investigated a possible effect of fentanyl exposure and naloxone administration on canine olfaction. Given that we often depend on these dogs’ olfactory capabilities not only to find hidden illicit drugs, but also for information from these searches to sustain scrutiny in a court of law, it is important to determine if fentanyl exposure and naloxone reversal affects canine olfaction (for further information, see [20]).

In the present study, we aimed to investigate the effects of fentanyl sedation and subsequent naloxone reversal on working dogs’ olfactory ability to locate a trained target odor. To investigate any differences in the route of naloxone administration, we used a crossover study in which sedated dogs were randomized to receive either intranasal or intramuscular naloxone and then following a washout period were again sedated and reversed by the other route.

## 2. Materials and Methods 

This protocol was approved by the Institutional Animal Care and Use Committee at the University of Pennsylvania for dogs owned by the University (Protocol #806379) and for the privately-owned dog (Protocol #806413).

### 2.1. Subjects

Dogs were eligible to enroll in the study if they were in training at (owned by the University) or graduates from the Penn Vet Working Dog Center (PVWDC), between 8 months and 7 years of age, and had been acclimated to handling required for catheter placement for sedation. In addition, eligible dogs must have been trained to detect Universal Detector Calibrant (UDC; [21], a synthetic odor not found in the natural environment) and be able to independently search the scent wheel. Dogs were excluded from the study if they did not meet the inclusion criteria, showed aggression or fear during restraint training, or had any medical condition that precluded sedation. Ten working dogs, nine of which were currently in-training at the PVWDC, and one that had graduated from the PVWDC as a narcotics detection dog participated (see Table 1). One of the dogs in-training at the PVWDC was in the process of being sold as a narcotics detection dog and had been trained on narcotics. All dogs lived with either their foster families (PVWDC dogs) or their handler (PVWDC graduate). All dogs had odor detection training at the PVWDC where they were trained to search for UDC and to give a final trained response (i.e., change of behavior alert such as stand-stare or sit-stare) to communicate to handlers that they have found the odor.

### 2.2. Scent Detection Methods

At the time of the study, all dogs were able to successfully find the lowest concentration of UDC available (48 mil with a 1.6 mm hole, dissipation rate = 0.27 ng/min) on a “scent wheel.” The dissipation rate of the odor is constant and predictable, therefore ensuring that the consistency in the detection difficulty between searches [21]. This stainless-steel scent wheel was 120 cm in diameter and contained 12 ports around the circumference. These ports held 29.5 mL glass jars 52 mm high and 38 mm diameter (SKS Bottle & Packaging, Stock #40210010.02S) used to contain items to place in the wheel. All glass jars were sterilized with 70% isopropyl alcohol prior to storing any odors used for scent detection. Ports were covered with 15 cm by 11 cm port covers with 3 mm wide gaps to keep the dogs from being able to come into contact with any items in the ports. The wheel was kept from moving during use by a brake placed underneath the wheel, which when pressed would allow the user to slowly spin the wheel, moving the location of all odors, either clockwise or counterclockwise. For the study, all dogs were tested on the scent wheel with one port containing UDC (48 mil with a 1.6 mm hole, dissipation rate = 0.27 ng/min), one port containing a blank-UDC powder control (what the UDC odor is impregnated on), and ten distractor odors the dogs would encounter throughout training (e.g., paper towel, glove). 

**Figure 1:**
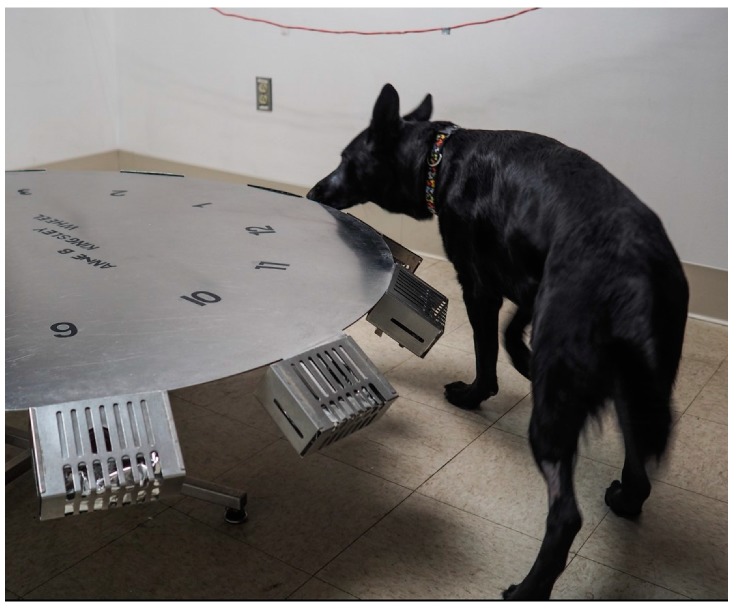
A dog searches the scent wheel—each port was numbered between 1 and 12 and held a different odor. These ports were then covered with metal grates to ensure the dogs could not come into contact with the odors.

**Figure 2:**
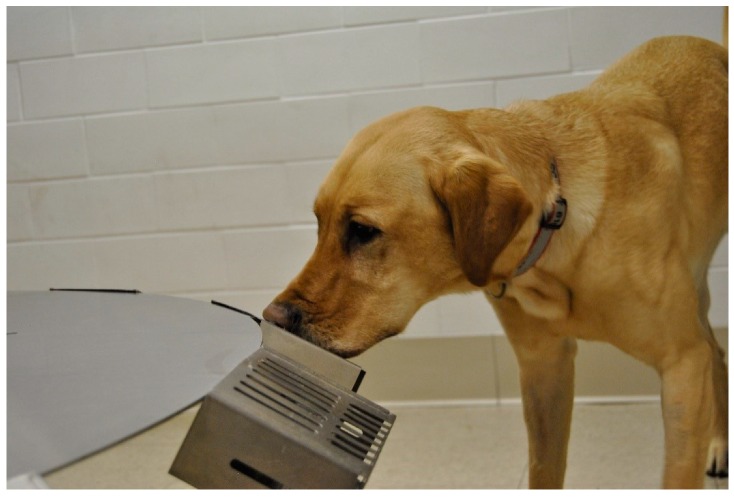
A dog giving a stand and stare alert on the port containing the target odor.

The location of the port in the room was not changed between trials on any day, so that no dog was at a disadvantage when entering the room regarding their initial proximity from the target odor. The location of the UDC (port number and location relevant to entrance) was randomized using a random number generator on each test day. Trials were blinded as dogs were sent onto the wheel independently and worked the wheel off-leash, out of sight of the handler (Figure 1). The handler was able to watch the dog work through a video connection into the wheel room in order to avoid influencing the search. Only one trial was carried out at each time point, where the dog was sent in and worked until they found the odor. When dogs successfully alerted on the UDC (Figure 2), the behavior would be marked with a click by the trainer (blind to the dog’s treatment status) and the dogs would come out of the wheel room to receive a reward, either a toy or a food reward depending on the dog’s preference. 

All searches were video recorded and later reviewed by a second individual to confirm the results. The scent wheel, including port covers, was cleaned with 70% isopropyl alcohol between each dog’s trial. These searches were carried out on the morning of the study prior to sedation event (“baseline”) and then 2, 24, and 48 h after sedation reversal. We anticipated that the sedation and reversal effects on olfaction, if present, would be apparent within 2 h of the procedure and wanted to monitor for resolution of any effect to know when dogs could effectively return to work. As we were interested in whether fentanyl sedation and naloxone reversal affected dogs’ ability to find their target odor, their behavior at the target odor port was marked as a false negative (FN), if they passed the target port or a true positive (TP), if they correctly alerted on the target port. We did not want to subjectively decide whether the dog was sniffing the target port as they passed, so we counted any physical pass of the target port by the dog as an FN. Dogs could pass the target port up to five times before being recalled from the room, with the trial ending (five passes being the standard used in our laboratory for odor detection trials).

The two dogs that were imprinted on narcotics were also evaluated on an independent and off-leash building search of four rooms approximately 6 by 6 m in size, which included various items inside such as shelving, tables, and cabinets. Each time point (e.g., pre-sedation, 2 h post-sedation, etc.) used different but similar sized and outfitted rooms. These timepoints were relative to the UDC searches, as they could not be done at the same time. All rooms were in the same building. At each time point, two of these rooms were empty, and two of these rooms contained narcotics (9 g marijuana in one room and 6.06 g heroin in the other room). Most hides were low to the ground (0–3 feet from the ground, N = 15), and one hide was slightly higher off the ground (4–6 feet from the ground, N = 1). These searches were double-blind, and the handlers were not influenced as to how to search other than which rooms to search. The handlers were not told when to leave or stay in a room; the handler or the dog made the decision to leave a room that was empty of odor. If a handler asked anything during the search, e.g., “I think we are done,” or “Is this room blank?”, no feedback was provided. These searches were also carried out on the morning of the study prior to sedation and then 2, 24, and 48 h after sedation reversal. This part of the study, though with only two dogs, was included to evaluate whether fentanyl sedation and naloxone reversal affected dogs’ ability to find narcotics odor, similar to a real-life drug search scenario.

### 2.3. Fentanyl Sedation and Naloxone Reversal

We carried out a cross-over design with a one-week washout between testing dates. The elimination half-life of fentanyl is 45.7 min [22] and naloxone is 71.2 min [23]. The seven-day washout provided a minimum of 140 elimination half-lives, and it was considered unlikely that either drug would have any residual effect. 

**Figure 3:**
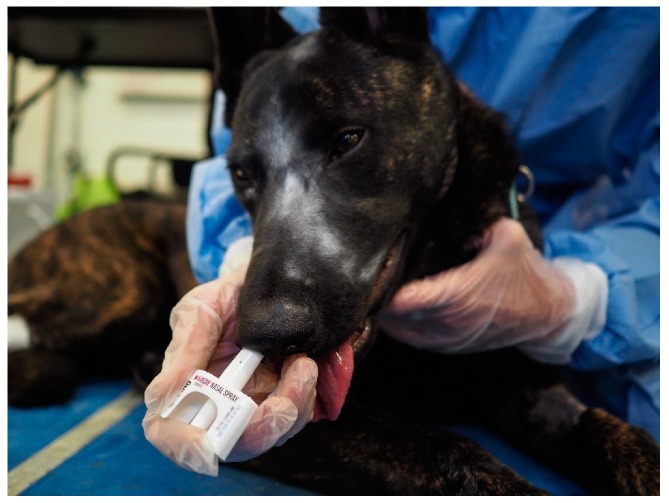
A fentanyl sedated dog receiving intranasal naloxone.

Dogs were block randomized by drawing names out of a hat, so that in the first week 5 dogs received intranasal (IN) and the remaining 5 dogs received intramuscular (IM) naloxone following sedation with intravenous fentanyl, then these groups switched for the second week. The order of sedation of the dogs each day was based on trainer preference. Dogs were sedated with 0.3 mg (0.05 mg/mL) of fentanyl citrate (Hospira, Inc., Lake Forest, IL, USA) IV. A blood sample was obtained for baseline naloxone concentrations following sedation. After 10 min, dogs were reversed based on random assignment to 4 mg of naloxone intranasally (0.1 mL Narcan; Adapt Pharma Inc, Radnor, PA, USA) in the right nostril (Figure 3) or 4 mg of intramuscular naloxone (4 mL, Naloxone, International Medication Systems, Limited, So., El Monte, CA) in the left lumbar epaxial muscle. Thus, dogs underwent this sedation protocol two times in total, once receiving intranasal naloxone and once receiving intramuscular naloxone.

Blood levels of naloxone and its metabolite, naloxol were analyzed by Liquid Chromatography Tandem Mass Spectrometry Analysis (LC-MS/MS) analysis, using a Sciex 6500 QTrap Triple Quadrupole Mass Spectrometer (Sciex, Ottawa, Ontario) coupled with an Agilent 1290 Infinity Liquid Chromatograph (Agilent Technologies, Santa Clara, CA, USA). Peak areas were integrated using the Analyst software (Sciex, Ottawa, Ontario, USA). The developed extraction method included a solid-phase absorption (SPE, Waters HLB, Milford, MA, USA) clean-up step followed by mass spectrometric analysis. Blank plasma from test subjects was obtained from the testing laboratory and was used to generate a 3-point inter-day method validation. This method was then applied to experimental samples. A calibration curve was generated each day that samples were analyzed to ensure the data was as accurate as possible. The Lower Limit of Detection was found to be 69.9 pg/mL for both naloxone and α-naloxol.

### 2.4. Scent Wheel

If a dog physically passed the target odor without exhibiting their trained final response, it was marked as a false negative (FN). If the dog alerted to a port other than the target odor, it was marked as a false positive (FP). A true positive (TP) was marked if the dog exhibited their trained final response at the port containing target odor. 

We analyzed two variables to investigate any effect of the treatment on the dogs’ olfaction: both whether or not they passed the target odor on their time approaching the odor (FN, Yes/No), and how many times they passed the target odor (Number of false negatives). For the first yes/no data, we ran a One Way Repeated Measures ANOVA with the dependent variable being the difference in frequency of false negatives between intranasal and intramuscular administration for each dog at each timepoint. For the dependent variable number of times each dog passed the target odor (FN), we ran a Two-Way Repeated Measures ANOVA with the independent variables of route of administration and timepoint. Both analyzed for an interaction between naloxone (intranasal or intramuscular administration) and timepoint (baseline, 2 h post, 4 h post, 24 h post, 48 h post). Residual naloxone or alpha naloxol were tested by comparing baseline blood concentrations after the first sedation (week 1) and the second sedation (week 2) using a One Way Repeated Measures ANOVA and a Friedman Repeated Measures Analysis of Variance on Ranks, respectively, All analyses were done in SigmaPlot for Windows, version 11.0 (Systat Software, San Jose, CA, USA). We considered *p* < 0.05 as significant.

### 2.5. Drug Searches

As we had only two dogs run drug searches, we report here only a summary of the results including whether the dog was successful in finding the narcotics and in leaving the empty control room(s). We are further able to report whether the dog “fringed” (or alerted near-to but not directly on the narcotics).

## 3. Results

Blood concentrations of naloxone and its metabolite, α-naloxol, were not different at the start of week 1 compared to week 2. See Table 2.

### 3.1. Scent Wheel

All dogs were able to find the lowest dissipation rate UDC (0.02 × 48 mil, dissipation rate = 0.27 ng/min) on the wheel at every timepoint, and the number of false alerts (FP) was extremely low (*N* = 2) across 80 trials. We did not find a statistically significant interaction between naloxone and timepoint for whether or not the dog passed their target odor at all (F = 0.669, *p* = 0.578), suggesting that neither intranasal nor intramuscular naloxone affected whether or not the dog passed their target odor (Table 3). We also did not find a statistically significant interaction between naloxone and timepoint for the number of times the dog passed their target odor (F = 0.499, *p* = 0.686), suggesting that neither intranasal nor intramuscular naloxone affected the number of times the dog passed their target odor at any timepoint post-administration (Table 3). All datapoints from the scent wheel analyses can be found in the Supplementary Materials (Table S1: Dataset).

### 3.2. Drug Searches

Dogs were successful at the drug searches at all time points, regardless of whether the dogs were reversed with IM (Table 4) or IN (Table 5) naloxone. Only one search was unsuccessful: Roxie was unable to find the heroin hide 48-h post-sedation, however, there were a lot of distractions in the area for this hide (see Discussion). All other searches were successful. We also had a low number of false alerts (alert on anything other than target odor, *N* = 2), and fringes (alert near but not directly on target odor, *N* = 1).

## 4. Discussion

We investigated whether intravenous (IV) fentanyl sedation and subsequent reversal with intranasal (IN) or intramuscular (IM) naloxone influenced canine olfaction. We found no evidence that dogs’ ability to find their target odor was influenced by either route of naloxone administration, from 2 h up to 48 h after sedation and reversal. Dogs were successful in searches in the laboratory-controlled setting (UDC) and in a building search (heroin and marijuana). Taken together, our results suggest there is no detectable effect of IV fentanyl sedation and either IN or IM naloxone reversal on the olfactory abilities of working dogs in this context.

One of the most difficult aspects of odor detection studies is controlling for differences between searches. Since we could ensure that the difficulty level of each search was the same by using UDC as our target odor and our scent wheel as the search apparatus, we have a high level of confidence that there was no tangible difference between searches across the time points each week. We kept the odor’s position on the wheel, and thus distance from the dog’s entrance to the room, consistent within testing days, so no dogs in any particular treatment would have a significantly easier (or harder) time finding their target odor. In this setting, we found that every dog was able to find the UDC target odor in every search, suggesting that any effect the fentanyl sedation and naloxone reversal may have had on the olfaction of the dogs was not substantial enough to keep the dogs from performing at these levels. However, the levels at which we could test the dogs on UDC were not at the threshold of dogs’ odor detection, which is presently unknown for this odor. Thus, while we did not see any effect on the dogs’ ability to find the UDC, it is still possible that there is some change in olfaction that is not captured within the scope this setup, and testing with lower-levels of UDC, closer to the threshold of the dogs, may find a subtle effect on olfaction.

We also found no evidence of an effect on the two dogs’ ability to find narcotics (heroin and marijuana) in typical room searches. Though heroin is illegal in all amounts at both the federal and state level, the amount of marijuana searched out by the dogs in this study (9 g) was lower than the amount that would result in a felony drug charge in states with the strictest laws on marijuana possession, for example Florida (20 g) and Tennessee (14 g) (excluding states which give felony drug charges for any amount of marijuana). The only failure to find target odor that we had was on one search, where Roxie was unable to locate the heroin hide 48 h after fentanyl sedation and intramuscular naloxone. We do not believe this was due to a change in her olfaction abilities, as her UDC detection was not similarly impaired, but rather due to the fact that many workers were present in the search location, unplanned, which introduced a high level of distraction for both dog and handler. Moreover, Scout was able to find the heroin in the same exact hide, and Roxie was able to find the heroin hide 48 h after the sedation and IN naloxone reversal. Thus, under the circumstances of this study, it is unlikely that the fentanyl sedation and naloxone reversal would have affected these dogs’ abilities to do their jobs as narcotics detection dogs.

Although the dogs that were enrolled in this study were capable of independently searching the scent wheel, they spent most of their training time searching for UDC and other target odors in room searches, rather than systematically searching the scent wheel. Thus, many of the dogs went in to the search and physically ‘passed’ the UDC (gave no alert) but were perhaps not searching the wheel directly. As we did not want to make subjective coding decisions on whether the dog did, or did not, actually sniff at each port, we counted all physical passes of the UDC as a ‘pass’ of the target odor. This was also especially relevant in two dogs, Ammo and Scout, who searched the wheel and room very quickly and, though they did continue to work to identify the source of the odor and found it in every trial (as did all dogs), they physically passed the UDC more frequently. Finally, most of the training and searching of these dogs on UDC in their daily training activities at the Center was on UDC of much higher dissipation rates than what we utilized in this study, so these searches were the most difficult searches the dogs had executed in terms of dissipation rate. 

Regarding the relevance of this study to the current risk of fentanyl exposure to working canines, we used IV fentanyl to induce sedation. This is an important difference as with IV fentanyl we were able to control the dosage, to ensure dogs received enough of the drug to induce sedation and allow us to subsequently reverse the sedation with naloxone, but without posing a serious risk to the dogs or personnel. Our study did not investigate the effects of fentanyl inhalation or mucous membrane absorption on odor threshold. However, the subsequent naloxone reversal (both IN and IM) was procedurally comparable to what would happen in the case of accidental fentanyl exposure and overdose in a canine in the field. 

## 5. Conclusions

We believe that this study presents the first report of any effects of fentanyl sedation and naloxone reversal on canine olfaction. Dogs were able to perform their searches for target odor at levels comparable to pre-sedation two-hours, 24 h, and 48 h post-sedation, including two dogs searching for narcotics, and irrespective of whether they received IN or IM naloxone reversal. Taken together, the results suggest that IV fentanyl sedation and subsequent IN or IM naloxone reversal do not affect dogs’ abilities to search out their target odors at the levels tested in this study.

## Figures and Tables

**Table 1:** Descriptive statistics of participant dogs. Sex “F” stands for female, while “M” stands for male. “UDC” stands for “Universal Detector Calibrant”.

Dog #	Name	Breed	Sex	Age (Months)	Status	Trained Odor(s)
1	DéjàVu	German Shepherd	F	13	In-Training	UDC
2	Ivey	German Shepherd	F	13	In-Training	UDC
3	Topper	German Shepherd	M	12	In-Training	UDC
4	Ellie	German Shepherd	F	10	In-Training	UDC
5	Max	German Shepherd	M	14	In-Training	UDC
6	Joey	Dutch Shepherd	M	8	In-Training	UDC
7	Lucy	Dutch Shepherd	F	8	In-Training	UDC
8	Ammo	Belgian Malinois	M	12	In-Training	UDC
9	Roxie	Labrador Retriever	F	43	In-Training	UDC, Narcotics
10	Scout	Labrador Retriever	M	31	Graduated	UDC, Narcotics

**Table 2:** Blood concentrations of naloxone and its metabolite, α-naloxol, at the start of weeks 1 and 2. * Values of zero were below the lower limit of detection (69.9 pg/mL).

Drug (ng/mL)	Week 1	Week 2
Naloxone	0.0 *	0.0 *
α-Naloxol	0.0 *	0.0 *

**Table 3:** Results for both ANOVA analyses for the scent wheel searches.

Analysis	Source of Variation	Degrees of Freedom	Sum of Squares	Mean Squares	F-Value	*p*-Value
Did the dog pass the target odor (Yes/No)?	Dog ID	9	29.063	3.229		
	Naloxone	1	0.613	0.613	0.310	0.591
	Naloxone x Dog ID	9	17.763	1.974		
	Timepoint	3	2.237	0.746	1.504	0.236
	Timepoint x Dog ID	27	13.387	0.496		
	Naloxone x Timepoint	3	0.638	0.213	0.499	0.686
	Residual	27	11.487	0.425		
	Total	79	75.188	0.952		
Number of times the dog passed the target odor	Between Subjects	9	5.225	0.581		
	Between Treatments	3	0.675	0.225	0.669	0.578
	Residual	27	9.075	0.336		
	Total	39	14.975			

**Table 4:** Summary of drug searches by dogs after receiving intramuscular naloxone.

Dog	Time	Successful Find			
		Heroin	Marijuana	False Alerts	Fringes
Scout	Pre-Sedation	Yes	Yes	0	0
	2 Hours Post Sedation	Yes	Yes	0	0
	24 Hours Post Sedation	Yes	Yes	0	0
	48 Hours Post Sedation	Yes	Yes	0	0
Roxie	Pre-Sedation	Yes	Yes	0	0
	2 Hours Post Sedation	Yes	Yes	0	0
	24 Hours Post Sedation	Yes	Yes	0	0
	48 Hours Post Sedation*	No **	Yes	1	1

* Workers were present during this search, resulting in a high level of distraction in the area. ** Medium-high hide (4–6 feet).

**Table 5:** Summary of drug searches by dogs after receiving intranasal naloxone.

Dog	Time	Successful Find			
		Heroin	Marijuana	False Alerts	Fringes
Scout	Pre-Sedation	Yes	Yes	0	0
	2 Hours Post Sedation	Yes	Yes	0	0
	24 Hours Post Sedation	Yes	Yes	0	0
	48 Hours Post Sedation*	Yes **	Yes	0	0
Roxie	Pre-Sedation	Yes	Yes	1	0
	2 Hours Post Sedation	Yes	Yes	0	0
	24 Hours Post Sedation	Yes	Yes	0	0
	48 Hours Post Sedation	Yes	Yes	0	0

* Workers were present during this search, resulting in a high level of distraction in the area. ** Medium-high hide (4–6 feet).

## Supplementary Materials

The following are available online at https://www.mdpi.com/2076-2615/9/6/385/s1, Table S1: Dataset.

## References

[1] Stanley T.H. (2014). The fentanyl story. J. Pain.

[2] Krinsky C.S., Lathrop S.L., Crossey M., Baker G., Zumwalt R. (2011). A toxicology-based review of fentanyl-related deaths in New Mexico (1986–2007). Am. J. Forensic Med. Pathol..

[3] Martin T.L., Woodall K.L., McLellan B.A. (2006). Fentanyl-related deaths in Ontario, Canada: Toxicological findings and circumstances of death in 112 cases (2002–2004). J. Anal. Toxicol..

[4] Frank R.G., Pollack H.A. (2017). Addressing the fentanyl threat to public health. N. Engl. J. Med..

[5] Wheeler E., Jones T.S., Gilbert M.K., Davidson P.J. (2015). Opioid overdose prevention programs providing naloxone to laypersons—United States, 2014. Morb. Mortal. Wkly. Rep..

[6] Palmer L.E., Gautier A. (2017). Clinical update: The risk of opioid toxicity and naloxone use in operational K9s. J. Spec. Oper. Med..

[7] Dahan A., Aarts L., Smith T.W. (2010). Incidence, reversal, and prevention of opioid-induced respiratory depression. Anesthesiology.

[8] Ngai S.H., Berkowitz B.A., Yang J.C., Hempstead J., Spector S. (1976). Pharmacokinetics of naloxone in rats and in man: Basis for its potency and short duration of action. Anesthesiology.

[9] Jezierski T., Adamkiewicz E., Walczak M., Sobczyńska M., Górecka-Bruzda A., Ensminger J., Papet E. (2014). Efficacy of drug detection by fully-trained police dogs varies by breed, training level, type of drug and search environment. Forensic Sci. Int..

[10] Patschke D., Eberlein H.J., Hess W., Tarnow J., Zimmerman G. (1977). Antagonism of morphine with naloxone in dogs: Cardiovascular effects with special reference to the coronary circulation. Br. J. Anaesth..

[11] Palminteri A. (1966). Clinical appraisal of the narcotic antagonist N-allylnoroxymorphone. J. Am. Vet. Med. Assoc..

[12] Lancaster K. (2014). Social construction and the evidence-based drug policy endeavour. Int. J. Drug Policy.

[13] Jezierski T., Ensminger J., Papet L.E. (2016). Canine Olfaction Science and Law: Advances in Forensic Science, Medicine, Conservation, and Environmental Remediation.

[14] Altom E.K., Davenport G.M., Myers L.J., Cummins K.A. (2003). Effect of dietary fat source and exercise on odorant-detecting ability of canine athletes. Res. Vet. Sci..

[15] Jenkins E.K., DeChant M.T., Perry E.B. (2018). When the nose doesn’t know: Canine olfactory function associated with health, management, and potential links to microbiota. Front. Vet. Sci..

[16] Hayes J.E., McGreevy P.D., Forbes S.L., Laing G., Stuetz R.M. (2018). Critical review of dog detection and the influences of physiology, training, and analytical methodologies. Talanta.

[17] Jenkins E.K., Lee-Fowler T.M., Angle T.C., Behrend E.N., Moore G.E. (2016). Effects of oral administration of metronidazole and doxycycline on olfactory capabilities of explosives detection dogs. Am. J. Vet. Res..

[18] Ezeh P.I., Myers L.J., Hanrahan L.A., Kemppainen R.J., Cummins K.A. (1992). Effects of steroids on the olfactory function of the dog. Physiol. Behav..

[19] Lien J. (2018). The Acute Effects of Isoflurane and Propofol on the Olfactory-Cognitive Ability of Brown Root Rot Disease Fungus Detection Dogs. Master’s Thesis.

[20] Scientific Working Group on Dog and Orthogonal Detector Guidelines. https://swgdog.fiu.edu/.

[21] Furton K.G., Caraballo N.I., Cerreta M.M., Holness H.K. (2015). Advances in the use of odour as forensic evidence through optimizing and standardizing instruments and canines. Philos. Trans. R. Soc. Lond. B. Biol. Sci..

[22] Sano T., Nishimura R., Kanazawa H., Igarashi E., Nagata Y., Mochizuki M., Sasaki N. (2006). Pharmacokinetics of fentanyl after single intravenous injection and constant rate infusion in dogs. Vet. Anaesth. Analg..

[23] Pace N.L., Parrish R.G., Lieberman M.M., Wong K.C., Blatnick R.A. (1979). Pharmacokinetics of naloxone and naltrexone in the dog. J. Pharmacol. Exp. Ther..

